# The Use of Volatile Substances in Drug-Facilitated Sexual Assault: A Systematic Review

**DOI:** 10.7759/cureus.33430

**Published:** 2023-01-05

**Authors:** Sadeem O AlOtaibi, Ghadi K Althinyan, Zainab A Alzakari, Farah A Mohamed, Ritesh G Menezes

**Affiliations:** 1 Department of General Medicine, College of Medicine, Imam Abdulrahman Bin Faisal University, Dammam, SAU; 2 Department of Pathology, College of Medicine, Imam Abdulrahman Bin Faisal University, Dammam, SAU

**Keywords:** violence against women and girls (vawg), violence against women, aromatic hydrocarbons, chloroform, volatile substances, date rape, drug-facilitated sexual assault

## Abstract

Drug-facilitated sexual assault (DFSA) is a significant crime that is increasing in incidence. The employment of volatile substances such as chloroform and aromatic petroleum hydrocarbons in DFSAs is quite an unusual choice. The objective of this review is to explore the use of volatile substances in DFSAs. Using the PubMed database, a systematic review of the literature was conducted. Thereafter, citation searching was carried out within the included studies from the primary search. A total of five studies were eligible for inclusion. Chloroform was the drug used in the DFSA in three of the included studies, and aromatic hydrocarbons in the remaining two. Two of the offenders who employed chloroform possessed a unique way to access the drug: their degrees. The evidence found in the DFSA cases included a chloroform-scented scarf and a solvent-immersed cloth. Headspace gas chromatography-mass spectrometry, liquid chromatography-electrospray coupled tandem mass spectrometry, toxicology assays of blood and urine, and solvent or hydrocarbon gas chromatography flame-ionization detection followed by gas chromatography-mass spectrometry were among the investigations performed to detect the volatile substances. The implementation of stricter regulations on chloroform for employees in chemical industries and laboratories is recommended. In cases where the autopsy is unclear and there are conspicuous facial and airway injuries, it is prudent to collect an early sample for volatile substance analysis.

## Introduction and background

Drug-facilitated sexual assault (DFSA) can be described as the use of drugs or alcohol to elicit unconsciousness in a victim for the purpose of nonconsensual sexual activity. Perpetrators can initiate sexual acts through either of the two types of DFSAs: proactive or opportunistic. Proactive DFSA occurs when victims ingest a substance that they are unaware of or that is forced upon them. Opportunistic DFSA, on the other hand, is the act of taking advantage of an intensely intoxicated person with a self-administered substance to actively initiate sexual activity [1]. DFSA represents a significant crime that is increasing in incidence, with most victims being females between the ages of 14 and 30 [2]. Cases remain quite underreported and underrepresented, partly because of the embarrassment and reluctance of friends and family who discover the assault but also because of fear of the offender. Additionally, the diagnosis of sexual assault may be evaded by physicians and only identified as a case of drug or alcohol intoxication [3-4]. Victims may reveal a wide variety of presentations, including amnesia, dizziness, confusion, decreased muscle control, impaired judgment, and bodily and genital injuries [5].

The preferred drugs used in DFSAs are commonly odorless and tasteless, making them the best choice for perpetrators. These drugs tend to easily dissolve in solvents, are rapidly absorbed via oral intake, and are effective at low doses. Importantly, these drugs are strong central nervous system depressants that can mimic critical alcohol intoxication. To conceal the drug and administer it without the victim’s knowledge, the perpetrator will use a vector such as alcoholic beverages or other drinks. In busy public places such as clubs or pubs, the perpetrators can easily slip these drugs into unattended drinks without the victim’s knowledge. Once the victim is sedated, the perpetrator may lead the incapacitated victim to a more private area for the purpose of sexual assault [6-7]. Alcohol is the most commonly administered substance due to the fact that it is considered fairly cheap, socially acceptable, and widely available. This is followed by benzodiazepines (BZD) [8-9]. Nonetheless, the literature reports a broad range of drugs, including antihistamines, anticholinergics, sedative-hypnotics, and opioids. The aforementioned drugs all share similar features to generate drowsiness with the incapacity to fight any sexual advances and anterograde amnesia; additionally, the properties of said drugs can be intensified when administered with alcohol. These drugs are usually ingested orally. Non-oral routes of administration have also been reported; inhaling volatile substances, such as chloroform and aromatic petroleum hydrocarbons, is one of the rare methods used to sedate the victim [10-11]. These compounds provoke the central nervous system's (CNS) depression and sedation like alcohol [12]. This form of DFSA is difficult to pursue without the victim’s knowledge of being assaulted, making it an unusual choice by a perpetrator [13]. Therefore, the aim of this systematic review is to analyze and explore the use of volatile substances for the purpose of DFSA.

## Review

Methods

This is a systematic review aiming to examine the use of volatile substances in DFSA.

Inclusion Criteria

The criteria used to select articles were as follows: (a) studies providing data on the employment of a volatile substance in DFSAs; (b) studies published in the English language; and (c) studies with primary data. A total of five studies were selected for this review, namely, three case reports, a case report and an experimental study, and a retrospective study.

Exclusion Criteria

Articles were excluded from this review based on the following criteria: (a) studies reporting on DFSAs in which no volatile substance was employed; (b) studies published in languages other than English; (c) review articles and thesis or conference paper abstracts; and (d) studies for which full text could not be obtained.

Search Strategy

The screening of titles and abstracts was carried out by four reviewers. Ultimately, each item was screened twice by two independent reviewers. The full text of studies that were deemed relevant was retrieved and then assessed for eligibility.

The primary search strategy for this systematic review is illustrated in Table 1. PubMed was used to search the literature on November 19, 2021. The search string used was as follows: (drug-facilitated sexual assault or date rape or rape drug) and (inhalation or inhalant or volatile or chloroform or aromatic or solvent or petroleum hydrocarbons or inhalation anesthetic or benzene or toluene or xylene), with no time limits applied. Thereafter, citation searching within the included studies from the primary search was conducted.

**Table 1:** Search strategy at PubMed

Search terms	Search details	Search results	Number of items that met the inclusion criteria
(drug-facilitated sexual assault OR date rape OR rape drug) AND (inhalation OR inhalant OR volatile OR chloroform OR aromatic OR solvent OR petroleum hydrocarbons OR inhalation anesthetic OR benzene OR toluene OR xylene)	(("Drug"[All Fields] AND "facilitated"[All Fields] AND "sexual"[All Fields] AND "assault"[All Fields]) OR ("date"[All Fields] AND "rape"[All Fields]) OR ("rape"[All Fields] AND "Drug"[All Fields])) AND ("inhalation"[Journal] OR "inhalation"[All Fields] OR "inhalant"[All Fields] OR "volatile"[All Fields] OR "chloroform"[All Fields] OR "aromatic"[All Fields] OR "solvent"[All Fields] OR ("petroleum"[All Fields] AND "hydrocarbons"[All Fields]) OR (("inhalation"[Journal] OR "inhalation"[All Fields]) AND "anesthetic"[All Fields]) OR "benzene"[All Fields] OR "toluene"[All Fields] OR "xylene"[All Fields])	104	4

Data Extraction

A checklist from Cochrane [14] was used to assist in data extraction from the articles that met the inclusion criteria. The extraction of data from each study was conducted independently by two reviewers, and the extracted data from the same article was compared afterward between the two reviewers. The extracted data were as follows: article title, authors, year of publication, geographical location, study design, and journal; profiling of the drug, victim, and offender; victim’s recollection; clinical and autopsy findings; investigations, including toxicological analysis and their results; and method of sample collection.

Results

Results of the Search Strategy

**Figure 1:**
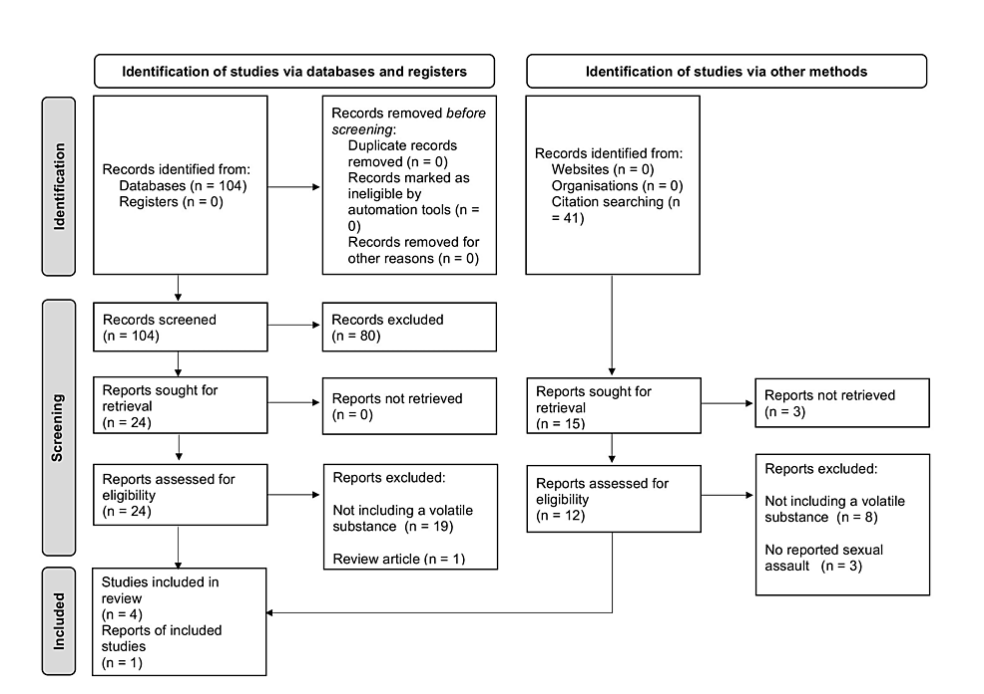
PRISMA flow diagram of study selection, which included searches of databases, registers, and other sources [18]

In total, 104 items were identified via the primary search through the PubMed database. Screening the titles and abstracts of identified items and records resulted in the exclusion of 80 items from the primary search. The remaining 24 records were sought for retrieval and assessed for eligibility. Four records from the PubMed search met the inclusion criteria. The primary search was followed by citation searching within the included studies from the PubMed database, identifying a total of 41 studies, of which one met the inclusion criteria. Finally, five studies [12-13,15-17] were included in this systematic review (Figure 1).

Profiling the Drug and Its Source

All the included studies are discussed here with regard to the drug being used, but only two (n=2) have reported on the source of the drug [13,16] (Table 2). 

Three of the included studies mention the use of chloroform for the purpose of DFSA [13,16,17]. Richeval et al. reported that chloroform is a controlled substance in France and is difficult to obtain. Therefore, it was suspected that the offender acquired the drug from a research laboratory as he was a Ph.D. student in chemistry [13]. Similarly, the offender in the case report by Gaillard et al. was speculated to have procured the chloroform from his company, since a 2.5-liter chloroform bottle was found opened at his workplace [16]. The two remaining studies included the use of aromatic hydrocarbons; the case report by Martínez et al. refers to the use of a combination of toluene, benzene, xylene, diazepam, and nordiazepam [12], while toluene alone was used in a single case of an alleged sexual assault found in the forensic service Northern Ireland (FSNI) database in the study reported by Hall et al. [15].

**Table 2:** Articles that met the inclusion criteria and volatile drugs used in DFSA DFSA: Drug-facilitated sexual assault

Title	Authors	Year of publication	Country/Continent	Journal	Study design	Drug	Source
An unusual case of drug-facilitated sexual assault using chloroform	Richeval et al. [13]	2016	France (Europe)	Journal of Analytical Toxicology	Case report	Chloroform	Offender’s research laboratory
Alleged drug-facilitated sexual assault (DFSA) in Northern Ireland from 1999 to 2005: a study of blood alcohol levels	Hall et al. [15]	2008	Northern Ireland (Europe)	Journal of Forensic and Legal Medicine	Retrospective study	Toluene	Not available
An unusual case of drug-facilitated sexual assault using aromatic solvents	Martínez et al. [12]	2006	Spain (Europe)	Journal of Analytical Toxicology	Case report	Toluene, Benzene, Xylene, Diazepam, and Nordiazepam	Not available
A case of drug-facilitated sexual assault leading to death by chloroform poisoning	Gaillard et al. [16]	2005	France (Europe)	International Journal of Legal Medicine	Case report	Chloroform	Offender’s workplace
Multiple homicides as a result of chloroform poisoning: a case report and experimental study	Risse et al. [17]	2001	Germany (Europe)	Forensic Science International	Case report and experimental study	Chloroform	Not available

Profiling the Offender

Two (n=2) out of the five included studies provided data on the identity of the offender [13,16] (Table 3).

Both offenders in the reports by Richeval et al. and Gaillard et al. had special access to chloroform due to their degrees, as one was a Ph.D. student in chemistry and the other was a 36-year-old male with a higher technical diploma in chemistry specializing in toxicology, respectively. Another similarity is that both cases had close contact with their victims; the first was the victim’s partner, and the second mentioned frequent close contact with the victim and her family. An additional important aspect was the presence of psychiatric implications, as the offender reported by Richeval et al. was hospitalized in a psychiatric department after the occurrence of the DFSA, whereas the offender reported by Gaillard et al. committed suicide within the same evening that the rape victim’s dead body was found [13,16]. The identities of the offenders in the remaining three studies were unknown. The case reported by Martínez et al. only mentions two individuals with their faces covered [12]. 

Profiling the Victim

Four (n=4) out of the five studies provided the victim’s sociodemographic characteristics [12-13,16-17] (Table 3).

Across all the studies that have reported on the victims' characteristics, the victims were all young females. The ages of the victims were as follows: a 26-year-old in the case by Richeval et al. [13], a 16-year-old in the report by Risse et al. [17], and a 13-year-old in the cases reported by Gaillard et al. and Martínez et al. [12,16].

**Table 3:** Characteristics of the offenders and victims DFSA: Drug-facilitated sexual assault

Reference	Age of the victim	Gender of the victim	Age of the offender	Gender of the offender	Employment/degree of the offender	Relationship with victim	Psychiatric implications in relation to the offender
Richeval et al. [13]	26-year-old	Female	Not available	Male	Ph.D. student in chemistry	Partner	Hospitalized in a psychiatric department after the DFSA
Hall et al. [15]	Not available	Not available	Not available	Not available	Not available	Not available	Not available
Martínez et al. [12]	13-year-old	Female	Not available	Not available	Not available	Not available	Not available
Gaillard et al. [16]	13-year-old	Female	36-year-old	Male	A higher technical diploma in chemistry specializing in toxicology	Close contact	Committed suicide on the same evening
Risse et al. [17]	16-year-old; 16-year-old	Female; Female	Not available	Not available	Not available	Not available	Not available

Evidence Found in the DFSA

Four (n=4) of the five studies reported on the evidence found in the DFSA [12-13,16-17] (Table 4). 

The victim in the case reported by Richeval et al. presumed aggression and sexual penetration after waking up with her hands tied and finding a scarf with the scent of chloroform. She was admitted to the hospital with complaints of back and cervical pain. She was examined thoroughly, and multiple signs were found, including several skin injuries, contusions on both wrists, cervical, scapular, and lumbar erosions, and ecchymoses and hematomas on the internal aspect of the legs and thighs. No injuries or significant signs were observed during gynecological, anal, and oral examinations [13]. In the case report by Gaillard et al., the victim was found dead in her bed, and an autopsy was performed afterward. The autopsy did not reveal any signs of injuries or violence, as evidence of violence was not found during the anal and vaginal examinations, considering the victim’s hymen was intact. An additional important aspect sought out during the autopsy was the absence of the chloroform odor and its corrosive effects [16]. One victim reported being kidnapped by two individuals with covered faces four hours prior to the sexual assault described in the report by Martínez et al. A cloth doused in liquid was placed over her mouth. The victim woke up afterward, semi-naked in the street, with a cell phone. She looked confused, and during the gynecological examination, no remarkable findings were observed [12]. In the case by Risse et al., the two victims were last seen in a discotheque. Later, their naked dead bodies were found at the edge of a path in a woodland; there was evidence of a sexual crime in both victims [17]. No details of the victim were provided in the study by Hall et al. [15].

**Table 4:** Evidence found in the DFSA cases

Reference	Victim (deceased/alive)	Victim’s recollection	Clinical findings	Autopsy findings
Richeval et al. [13]	Alive	Tied hands and a scarf with the scent of chloroform, raising suspicion of aggression and sexual penetration.	Cervical and back pain, skin injuries, contusions in both wrists, cervical, scapular, and lumbar erosions, ecchymoses and hematomas on the internal aspect of the thighs and legs, unremarkable gynecological, oral, and anal examinations	Not applicable
Hall et al. [15]	Not available	Not available	Not available	Not applicable
Martínez et al. [12]	Alive	Kidnapped by two individuals with covered faces four hours prior to the sexual assault. A cloth doused in a solvent was placed on her mouth. The victim woke up afterward in the street, semi-naked, with a cell phone.	The victim looked confused. Unremarkable gynecological examination.	Not applicable
Gaillard et al. [16]	Deceased	Not available	Not applicable. The victim was found dead on her bed.	No signs of injuries or violence. Unremarkable anal and vaginal examination. Negative chloroform odor and its corrosive effects on bodily tissues.
Risse et al. [17]	Deceased	Not available	Not available	Not available

Investigations Including Toxicological Analysis

All included studies are discussed in this section [12-13,15-17] (Table 5).

In the case report by Richeval et al., headspace gas chromatography-mass spectrometry (HS-GC/MS) and liquid chromatography-electrospray coupled tandem mass spectrometry methods were performed to detect drugs in the victim’s blood and the chloroform-scented scarf. The HS-GC/MS method detected the presence of chloroform in the blood, with a concentration of 580 micrograms/L, and in the scarf. Other tests, including the prostate-specific antigen (PSA) test, pregnancy test, complete blood count (CBC), electrolytes, and coagulation profile, were performed and were all unremarkable [13]. Gaillard et al. reported that HS-GC/MS was performed in both the victim's and the offender's samples. The chloroform concentration in the victim’s peripheral blood was 833.9 mg/L, whereas the offender’s cardiac blood concentration was 0.25 mg/L. Noticeably, the concentrations in the offender’s kidneys and lungs were 0.34 mg/kg and 0.30 mg/kg, respectively, as well as a concentration of 5.44 mg/kg in the subcutaneous fat. The presence of sperm was noted in the victim’s vaginal swab. In addition, the DNA was matched with the offender’s using the seminal fluid for DNA detection. Additional investigations were performed, including the immunoassay technique and carbon monoxide and cyanide detection using blood, bile, urine, and hair samples [16]. The case report by Martínez et al. mentions that solvent/hydrocarbon gas chromatography flame-ionization detection followed by gas chromatography-mass spectrometry (GC-FID/GC-MS) was performed, which detected benzene (7.6 mg/L), toluene (24.8 mg/L), and xylene (0.6 mg/L). Acidic-neutral and basic drug screening of the blood with gas chromatography with nitrogen-phosphorus detection and gas chromatography-mass spectrometry (GC-NPD/GC-MS) after solid-phase extraction (SPE) was also performed and found small amounts of diazepam (0.02 mg/L) and nordiazepam (below the limit of quantification). Headspace with GC-FID was performed and was negative for ethanol and volatiles such as sevoflurane, chloroform, halothane, and isoflurane; an immunoassay of the blood was negative for illicit and therapeutic drugs as well [12]. In the study conducted by Hall et al., a toxicology assay of the victim’s blood and urine was carried out, which revealed the presence of toluene [15]. In the case by Risse et al., only HS-GC/MS was performed, the results of which revealed a blood concentration of chloroform of 86 in one victim and 115 mg/L in another victim [17].

**Table 5:** Investigations performed in DFSA cases with volatile substances CBC: complete blood count; HS-GC-FID: headspace gas chromatography with flame ionization detection; LC-ESI-MS/MS: liquid chromatography-electrospray ionization–tandem mass spectrometry; GC-NPD: gas chromatography with nitrogen-phosphorus detection; GC-MS: gas chromatography with mass spectrometry; SPE: solid-phase extraction; LC-PDA: liquid chromatography photodiode array; HS-GC-MS: headspace gas chromatography with mass spectrometry; LC-MS-MS: liquid chromatography with tandem mass spectrometry; DNA: deoxyribonucleic acid

Reference	Investigations	Results
Richeval et al. [13]	Prostate-specific antigen	Negative
	Pregnancy test	Negative
	Coagulation parameters	Unremarkable
	CBC and electrolytes	Unremarkable
	Infectious disease status	Unremarkable
	HS-GC-FID, LC-ESI-MS/MS	Chloroform positive in blood concentration of 580 *μg*/L, scarf
Hall et al. [15]	Toxicology assay of blood and urine	Positive for toluene
Martínez et al. [12]	HS-GC-FID	Negative
	Immunoassay of the blood	Unremarkable
	Acidic-neutral and basic drug screening with GC-NPD/GC-MS after SPE extraction	Small amounts of diazepam (0.02 mg/L) and nordiazepam (below the limit of quantification)
	Solvent/hydrocarbon GC-FID/GC-MS	Benzene (7.6 mg/L), toluene (24.8 mg/L), and xylene (0.6 mg/L)
Gaillard et al. [16]	Immunoassay technique	Unremarkable
	Carbon monoxide detection	Negative
	Cyanide detection	Negative
	GC-MS, LC-PDA, HS-GC-MS, LC-MS-MS	Chloroform positive in; a) Victim: peripheral blood concentration of chloroform 833.9 mg/L; b) Offender: cardiac blood concentration of 0.25 mg/L, kidney concentration of 0.34 mg/kg, lung concentration of 0.30 mg/kg, subcutaneous fat 5.44 mg/kg
	Vaginal swab	Positive for sperm DNA detection of seminal fluid matching the offender.
Risse et al. [17]	HS-GC-MS	Chloroform positive in; a) Victim 1: blood concentration of chloroform was 86 mg/L; b) Victim 2: blood concentration of chloroform was 115 mg/L

Method of Sample Collection

A single study (n=1) out of the five studies is discussed here [17].

In the experimental study of Risse et al., it was reported that using a glass tube with a ground glass stopper to collect samples was superior to using a polyethylene tube, as the latter was associated with additional losses of chloroform. In addition, in the case of an equivocal autopsy, it was advisable to obtain an additional blood sample for volatile substances only; this way, the losses that occur with repeated opening of the tubes in the process of testing for other substances are prevented. The additional sample is best stored at freezing temperatures (-20 °C) [17].

Discussion

This systematic review has analyzed five different studies of DFSA using volatile substances. The characteristics of the victim, the offender, and the drugs used; the victim’s recollection; clinical and autopsy findings; investigations, including toxicological analysis performed with their results; and the method of sample collection were all explored.

Chemical submission can be defined as the administration of substances without the victim’s awareness to produce incapacitation and passivity to initiate drug-facilitated crimes (DFC) such as robberies or homicides and DFSAs [19]. Throughout all the included studies, two groups of drugs have been mentioned: chloroform and aromatic hydrocarbons (benzene, toluene, and xylene). Chloroform and aromatic hydrocarbons are considered unusual choices of drugs for the purpose of DFSAs. The preferred drugs tend to be odorless, tasteless, and easily dissolved; hence, victims ingest the dosed beverages unsuspectingly [6]. In contrast, the use of volatile substances in a DFSA cannot be pursued without the victim’s awareness of being assaulted [13]. The mode of administration of these substances requires covering the victim’s airway with a cloth doused in the solvent; therefore, the victim is completely aware of the situation, making it difficult for the offender to oppose the victim’s defenses [12]. Chloroform was initially utilized to induce and maintain medical anesthesia; however, with further research, it was proven to cause hepatotoxicity. With the rise of better and safer drugs, the clinical use of chloroform has been discontinued [20]. Today, due to its strict regulations, the accessibility of pure chloroform is restricted. However, the intentional inhalation of chloroform to commit or attempt drug-facilitated crimes remains present [21]. In both case reports by Richeval et al. and Gaillard et al., the DFSA took place in France, where chloroform is a controlled substance. In addition, the offender in the report by Richeval et al. was a Ph.D. student in chemistry, and in the report by Gaillard et al., the offender had a higher technical diploma in chemistry specializing in toxicology. Therefore, both had easy access to chloroform. In the first case, the offender retrieved it from a research lab, and in the second case, the offender acquired it from his workplace [13,16].

Chloroform has been reported in the literature for its use in several types of drug-facilitated crimes (DFCs), including homicides, robberies [22], and sexual assaults. The voluntary inhalation of chloroform as a mode of suicide has also been documented in forensic studies [23]. Aromatic hydrocarbons are found in cheap and easily available products, such as paint thinners, acrylic spray paints, cleaning products, gasoline, nail polish, hair dye, airplane glue, and plastic cement. These abundantly available volatile substances are consumed via inhalation, which can give rise to substantial toxicity [24]. People may come into contact with these substances through several methods at home, at their place of work, or through deliberate inhalation, as they are misused frequently for their rapid central nervous system (CNS) effects [25]. The use of aromatic hydrocarbons was reported in two of the included studies. Martínez and Ballesteros’ case report mentioned the utilization of a combination of benzene, toluene, and xylene, while Hall et al. reported the employment of toluene alone [12,15]. Aromatic hydrocarbons have been correlated with volatile substance abuse through the inhalation of these substances. Products such as glue that contain aromatic hydrocarbons have been frequently cited as the choice of substance for abuse and, in many cases, have led to death, such as in two cases of death due to toluene intoxication because of glue inhalation [26-27]. Fatal intoxication by aromatic hydrocarbons is also more commonly seen in cases of accidental and occupational inhalation, including several cases of fatal accidental gasoline inhalation as well as paint thinner toxicosis with lethal concentrations of toluene [28-31].

Limitations

A limitation that was perceived during the conduct of this systematic review was relying on a single database, as this study was achieved using PubMed for literature search. Using additional databases may help achieve better insight and understanding of this important topic.

## Conclusions

Worldwide, chloroform is a regulated substance due to its known toxicity, yet the current review shows how easily accessible it was to workers in chemical industries and laboratories, demonstrating the potential need for stricter regulations for those who work in these fields. In contrast, aromatic hydrocarbons are more widely available to the general community, and accordingly, it would be more challenging to enforce regulations on a global scale. Uncertain autopsy cases with conspicuous facial and airway lesions are highly suggestive of inhalation of volatile substances; thus, early sample collection for analysis of volatile substances is advisable to decrease losses. Although we have found only five studies reporting on the use of volatile substances in DFSAs, the increasing number of underreported cases of sexual assault is a global concern. Therefore, it is difficult to presume that DFSAs involving volatile substances are a less common act. Future research addressing the employment of volatile substances in DFSAs is recommended.
